# Targeting MET kinase with the small-molecule inhibitor amuvatinib induces cytotoxicity in primary myeloma cells and cell lines

**DOI:** 10.1186/1756-8722-6-92

**Published:** 2013-12-10

**Authors:** Cornel Joseph Phillip, Shadia Zaman, Shujun Shentu, Kumudha Balakrishnan, Jiexin Zhang, Veera Baladandayuthapani, Pietro Taverna, Sanjeev Redkar, Michael Wang, Christine Marie Stellrecht, Varsha Gandhi

**Affiliations:** 1 Departments of Experimental Therapeutics, The University of Texas MD Anderson Cancer Center, Houston, Texas, USA; 2 Bioinformatics and Computational Biology, The University of Texas MD Anderson Cancer Center, Houston, Texas, USA; 3 Biostatistics, The University of Texas MD Anderson Cancer Center, Houston, Texas, USA; 4 Leukemia, The University of Texas MD Anderson Cancer Center, Houston, Texas, USA; 5 Lymphoma/Myeloma, The University of Texas MD Anderson Cancer Center, Houston, Texas, USA; 6 Graduate School of Biomedical Sciences, The University of Texas Health Science Center, Houston, Texas, USA; 7 Astex Pharmaceuticals, Inc., Dublin, California, USA

**Keywords:** MET, HGF, amuvatinib, MP470, Multiple myeloma

## Abstract

**Background:**

MET is a receptor tyrosine kinase that is activated by the ligand HGF and this pathway promotes cell survival, migration, and motility. In accordance with its oncogenic role, MET is constitutively active, mutated, or over-expressed in many cancers. Corollary to its impact, inhibition of MET kinase activity causes reduction of the downstream signaling and demise of cells. In myeloma, a B-cell plasma malignancy, MET is neither mutated nor over-expressed, however, HGF is increased in plasma or serum obtained from myeloma patients and this was associated with poor prognosis. The small-molecule, amuvatinib, inhibits MET receptor tyrosine kinase. Based on this background, we hypothesized that targeting the HGF/MET signaling pathway is a rational approach to myeloma therapy and that myeloma cells would be sensitive to amuvatinib.

**Methods:**

Expression of MET and HGF mRNAs in normal versus malignant plasma cells was compared during disease progression. Cell death and growth as well as MET signaling pathway were assessed in amuvatinib treated primary myeloma cells and cell lines.

**Results:**

There was a progressive increase in the transcript levels of HGF (but not MET) from normal plasma cells to refractory malignant plasma cells. Amuvatinib readily inhibited MET phosphorylation in primary CD138+ cells from myeloma patients and in concordance, increased cell death. A 48-hr amuvatinib treatment in high HGF-expressing myeloma cell line, U266, resulted in growth inhibition. Levels of cytotoxicity were time-dependent; at 24, 48, and 72 h, amuvatinib (25 μM) resulted in 28%, 40%, and 55% cell death. Consistent with these data, there was an amuvatinib-mediated decrease in MET phosphorylation in the cell line. Amuvatinib at concentrations of 5, 10, or 25 μM readily inhibited HGF-dependent MET, AKT, ERK and GSK-3-beta phosphorylation. MET-mediated effects were not observed in myeloma cell line that has low MET and/or HGF expression.

**Conclusions:**

These data suggest that at the cellular level MET/HGF pathway inclines with myeloma disease progression. Amuvatinib, a small molecule MET kinase inhibitor, is effective in inducing growth inhibition and cell death in myeloma cell lines as well as primary malignant plasma cells. These cytostatic and cytotoxic effects were associated with an impact on MET/HGF pathway.

## Introduction

Multiple myeloma (MM) is an indolent B-cell disease that develops in the bone marrow and is associated with osteolytic lesions in the advanced stages [1]. Despite progress in prolonging myeloma patient survival, current therapies are not curative; thus, it is imperative that new treatments be developed for this debilitating disease [2,3].

Survival and proliferation of myeloma cells are dependent on the presence of a permissive microenvironment, which includes bone marrow stroma and soluble cytokines [4-9] such as IL-6 and HGF [8,10]. HGF is the ligand for MET receptor tyrosine kinase. When HGF binds to and activates MET, MET is autophosphorylated on Tyr1230, Tyr1234 and Tyr1235 located in the activation loop [11-14]. In addition, MET has a multisubstrate docking site that is activated at Tyr1349 and Tyr1356. The phosphorylation of this region results in the induction of MET signaling through the activation of several downstream target pathways, including the mitogen-activated protein kinase (MAPK) and AKT signaling pathways [11]. HGF/MET-induced MAPK signaling has been shown to be essential for proliferation, migration and invasion [7,11,15,16] while the induction of AKT signaling promotes tumor cell survival [17].

HGF/MET signaling is increasingly recognized as an important contributor to the pathogenesis of myeloma. Expression of both HGF and MET has been demonstrated in most myeloma cell lines and primary patient samples [18,19]. Studies correlating HGF levels with MM clinical parameters such as diagnosis [20-23] disease stage, aggressiveness [22,24,25], prognosis [22,23,26], and response [26-29]. Besides its effects on the malignant myeloma cells, HGF is involved in the pathogenesis of myeloma-related bone disease. HGF levels are increased in patients with extensive bone lesions, and correlates with expression of osteoclast stimulating cytokines [24]. IL-11 secretion from osteoblasts is induced by HGF [30], and HGF inhibits bone morphogenetic protein-induced osteoblastogenesis [31].

Taken together, these clinical findings strongly support our hypothesis that targeting the HGF/MET signaling pathway is a rational approach to myeloma therapy. In line with this postulate, our laboratory studies demonstrated that genetically knocking down MET in myeloma cell lines using short hairpin RNA and ribozyme approaches resulted in growth inhibition and demise of the myeloma cells [32,33]. Consistent with these observations, a decline in MET transcript and protein levels induced by treatment with any of the transcription inhibitors flavopiridol, cordycepin, or 8-chloro-adenosine, promoted myeloma cell death [32-34]. Collectively, these data demonstrate MET’s pivotal role in myeloma cell biology and underscore the importance of MET targeting as a therapeutic strategy in MM [35].

While these genetic and pharmacologic strategies suggest utility of MET/HGF inhibition as therapeutic targets, these interventions are not pragmatic for clinical use. Amuvatinib (previously known as MP470, Astex Pharmaceuticals, Inc.) is a synthetic carbothioamide that inhibits MET, cKIT and platelet derived growth factor receptor (PDGFR). This small-molecule inhibitor competes with ATP for binding at the catalytic site. In solid cancers, amuvatinib has been shown to be effective in inhibiting MET at low micromolar concentrations (IC_50_ ~5 μM) [36]. Amuvatinib is a well-tolerated, orally bioavailable drug currently in phase II clinical trials [37,38]. Availability of a clinical candidate, its inhibitory potential for MET kinase, and the role of MET in myeloma cell survival provided compelling rationales for testing the effects of amuvatinib on myeloma cells.

In the present study, we compared mRNA levels of *MET* and *HGF* in normal and primary myeloma plasma cells. We investigated amuvatinib’s actions and cytotoxic effects in primary plasma cells obtained from patients with myeloma. To elucidate in more detail the mechanism of action of amuvatinib in myeloma cells, we evaluated its effect on MET activity and downstream signaling in the myeloma cell line U266, which over-expresses HGF. Our data demonstrate that MET receptor tyrosine kinase may be targeted in myeloma and support the investigation of small-molecule inhibitors such as amuvatinib as possible therapeutic agents against this disease.

## Results

### Expression levels of *MET* and *HGF* mRNA in bone marrow plasma cells of healthy donors and patients

Previously studies have correlated plasma HGF levels with MM clinical parameters such as diagnosis [20-23] disease stage, aggressiveness [22,24,25], prognosis [22,23,26], and response [26-29]. While expression of both *HGF* and *MET* transcripts has been shown to be present in myeloma cells [18,19] and *HGF* mRNA has also been demonstrated to be expressed in bone marrow stromal cells [39] the levels of *HGF* and *MET mRNA* in patient plasma cells have not been well evaluated nor correlated with disease status.

**Figure 1:**
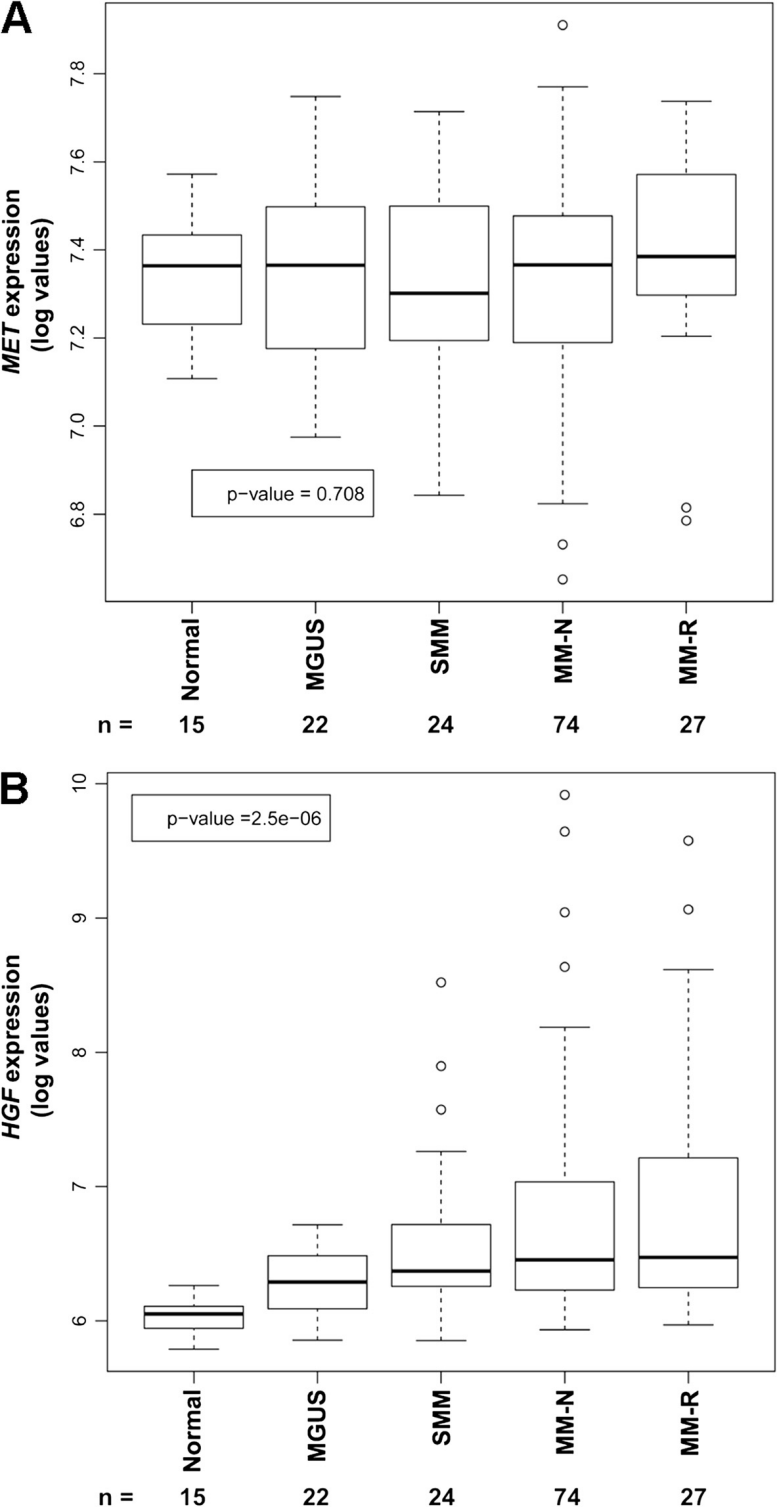
**Expression levels of*HGF*mRNA, but not*MET*, in CD138+ cells increase progressively from healthy individuals to myeloma patients.** Publically available expression array data was Robust Multichip Average processed and plotted as boxplots of relative **(A)***MET* and **(B)***HGF* mRNA log expression values in healthy donors (normal), and in patients with monoclonal gammopathy of undetermined significance (MGUS), smoldering multiple myeloma (SMM), newly-diagnosed multiple myeloma (MM-N), or relapsed/refractory disease (MM-R). Boxplots show 25th and 75th percentiles and the bold lines as medians. Whiskers show the range of values within the interquantile range from the box, and circles are outliers. Numbers at the bottom of each graph indicate the number of individuals in each category.

To determine the levels of MET and HGF gene expression in malignant and normal plasma cells, we analyzed data from the Mayo Clinic Patient Dataset available in the public domain [40,41]. The 162 samples evaluated represented 15 healthy individuals (normal), 22 patients with monoclonal gammopathy of undetermined significance (MGUS), 24 with smoldering MM (SMM), 74 with newly diagnosed MM (MM-N), and 27 with relapsed/refractory MM (MM-R). Among these five groups, there was no significant difference (*P* = 0.708) in the expression of *MET* in the CD138+ cells (Figure 1A). In contrast, there was a significant trend (*P* = 2.5 × 10^-06^) for increases in *HGF* mRNA levels in CD138+ plasma cells, with progressive severity of disease from healthy donors to patients with relapsed or refractory MM (Figure 1B). Within each group, there was heterogeneity in *HGF* expression as evinced by the 75th percentile mark. It is interesting to note that even samples with lower *HGF* mRNA levels in the plasma cells, typically had higher levels than the samples from healthy individuals; the 25th percentile for the myeloma patient *HGF* levels was > the 75th percentile for healthy individuals.

### Induction of apoptosis by amuvatinib in primary CD138+ and CD138– Cells

**Figure 2:**
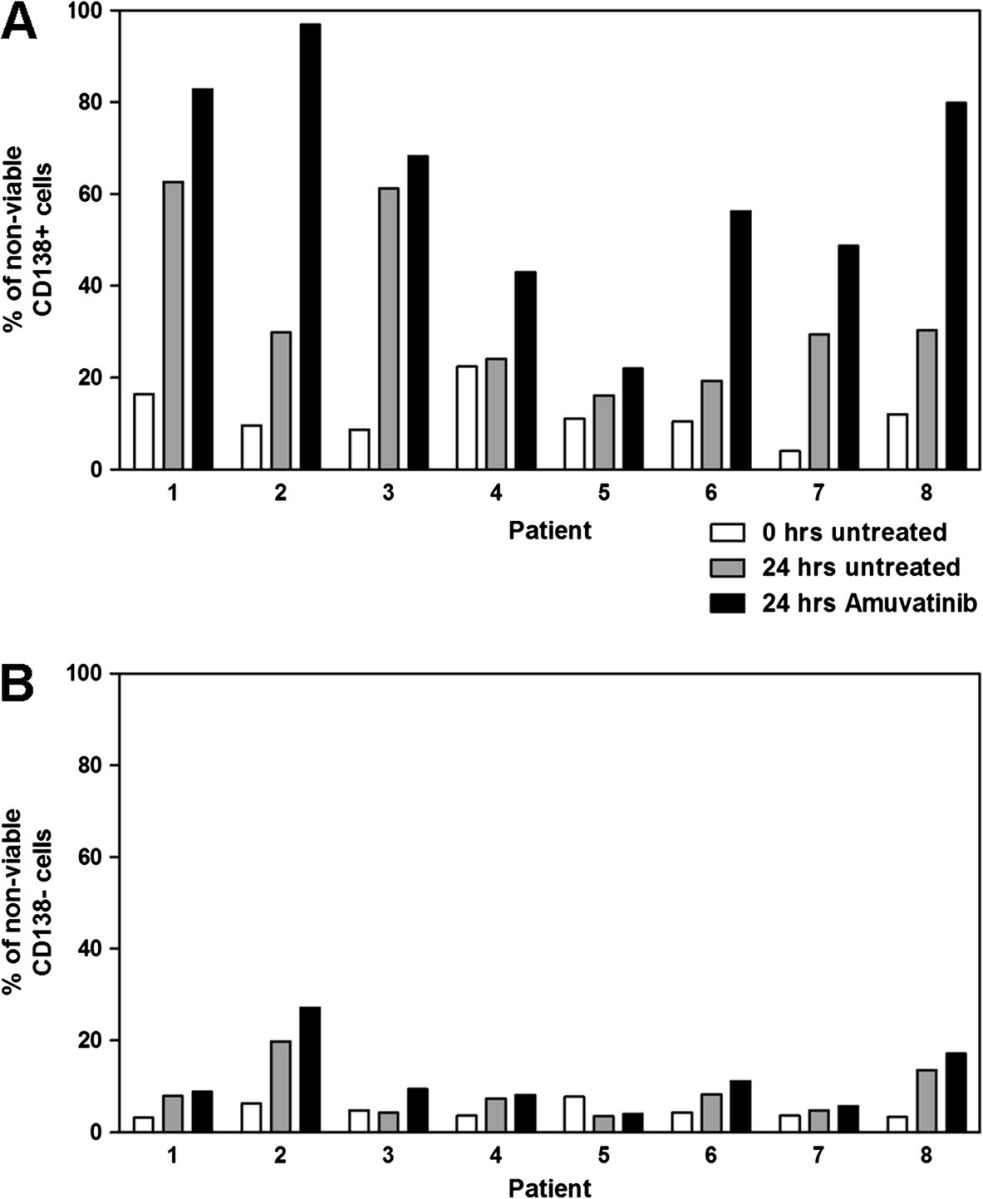
**Amuvatinib induces apoptosis in CD138+, but not CD138– cells. (A)** CD138+ and **(B)** CD138– cells isolated and purified from bone marrow samples of eight myeloma patients were stained with annexin V/PI and assessed by flow cytometry initially after isolation (white bars) or after 24 h of treatment with vehicle (gray bars) or 25 μM amuvatinib (black bars).

Gene array analysis along with numerous other studies by us [32,33] and others [22,23,26], identified the HGF/MET axis as a therapeutic target in myeloma. To test this, we assessed the sensitivity of primary myeloma cells to the MET-kinase inhibitor, amuvatinib. CD138+ (myeloma plasma cells) and CD138– (non-malignant cells) cells were isolated from bone marrow samples obtained from eight myeloma patients (Table 1) and treated with 25 μM amuvatinib for 24 h. This dose was chosen based on findings that >95% of the compound is bound and sequestered by serum proteins (unpublished data) as well as a small preliminary screen in myeloma cell lines (data not shown). CD138+ cells from six out of eight patient samples showed a cell death induction in the amuvatinib treated cells compared to time-matched dimethyl sulfoxide (DMSO)-treated control cells as measured by annexin V/propidium iodide (PI) staining (Figure 2A). Cell death in these six samples increased by 20% to 67% compared to time-matched controls; patients 3 and 5 showed <10% increase in cell death.

**Table 1:** Myeloma patient characteristics

**Pt #**	**Age (yrs)**	**Sex**	**Ethnicity**	**WBC**^***a ***^**(10**^**3**^**/μl)**	**Plasma cells (%)**	**BM**^***b ***^**aspirate done**	**Outcome of treatment**
**1**	62	Male	White	3.6	7	At diagnosis	NA^***c***^
**2**	53	Female	Black	2.0	74	Post bortezomib + doxorubicin + lenalidomide + dexamethasone	Progressive
**3**	53	Female	White	6.3	4	Post 3 cycles bortezomib + dexamethasone	Near complete remission
**4**	76	Female	White	4.4	2	Post cyclophosphamide + lenalidomide + dexamethasone	Progressive
**5**	58	Female	Hispanic	9.1	8	At diagnosis	NA
**6**	64	Female	White	12.5	49	At diagnosis	NA
**7**	71	Male	White	3.9	32	NA	NA
**8**	76	Male	White	4.9	36	post 1 yr auto stem cell transplant	Progressive

^***a***^white blood cells; ^***b***^bone marrow; ^***c***^not applicable.

An assessment of clinical characteristics of the myeloma patients did not reveal any correlations with amuvatinib-induced cell death, including prior treatment, patient age, or cellularity of the bone marrow (Table 1). We were able to measure the HGF levels in plasma samples from patients 2, 4, and 5 which were 11.9, 1.7, and 1.4 ng/mL, respectively. Although the number of total samples is small, there seems to be a relationship between levels of HGF and amuvatinib-induced apoptosis. Additional studies are needed to determine if there is any correlation between HGF level and sensitivity of CD138+ cells to MET inhibition.

In contrast to the sensitivity of CD138+ cells to amuvatinib, CD138– cells did not show any sizeable inductions (all, <10%) of death compared to time-matched controls (Figure 2B), suggesting that non-malignant bone marrow cells are not affected by amuvatinib. Overall, these results indicate that treatment of CD138+ MM cells with a MET inhibitor is detrimental to their survival.

### Effect of Amuvatinib on MET signaling in CD138+ and CD138- cells

**Figure 3:**
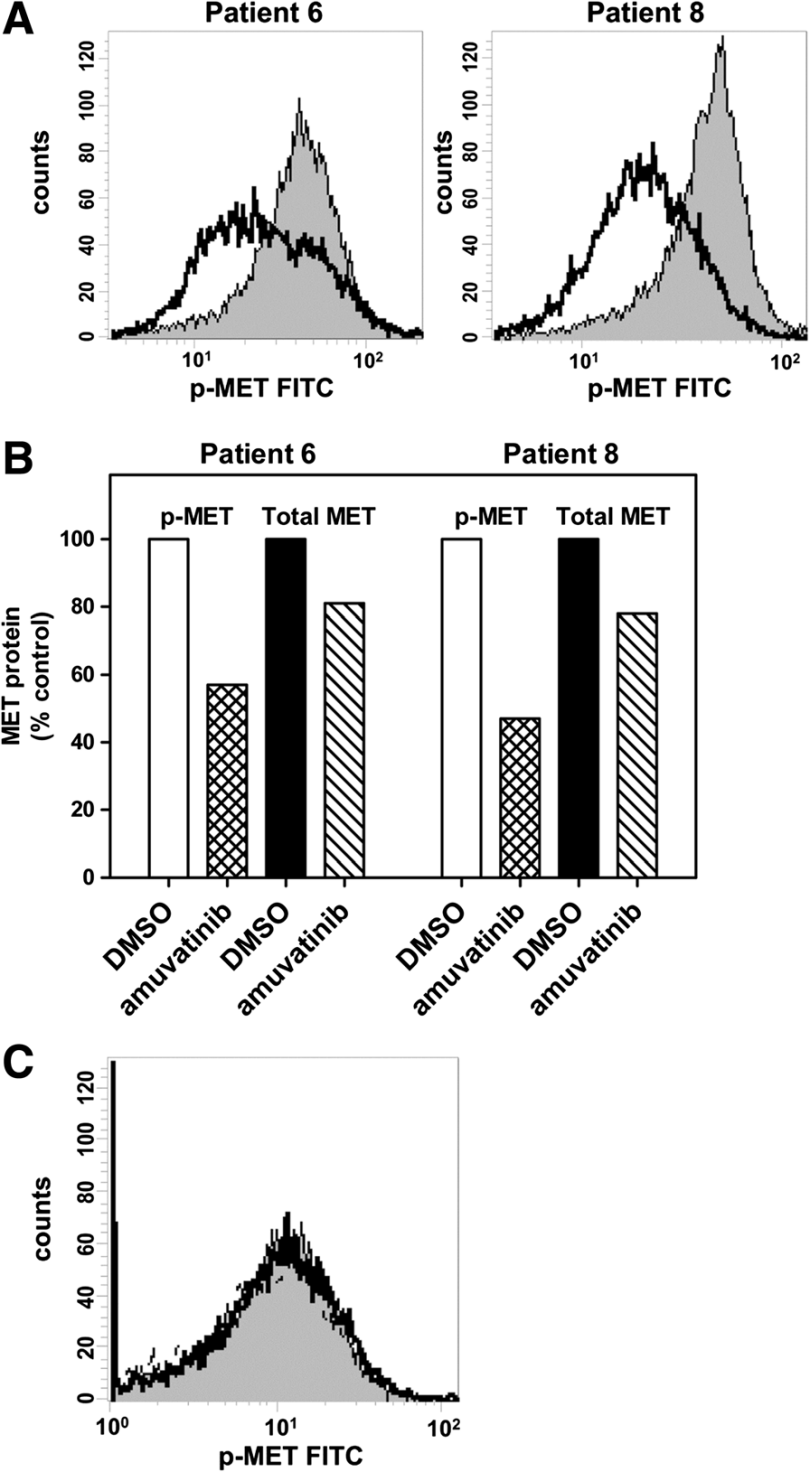
**Amuvatinib treatment decreases phospho-MET protein in freshly obtained CD138+ cells. (A)** Flow cytometric analysis of the levels of p-MET (Tyr1234/Tyr1235) in CD138+ cells from patients 6 and 8 after 24 h treatment with (thick black line) or without (gray shaded peak) 25 μM amuvatinib. **(B)** p-MET and total MET were quantified in untreated (white bars) and 25 μM amuvatinib treated cells (hashed bars) from panel **(A)** along with total MET levels in untreated (black bars) and 25 μM amuvatinib treated cells (diagonal bars). **(C)** Plot represents the levels of p-MET (Tyr1234/Tyr1235) in CD138– cells from patient 8 24 h after treatment with (thick black line) or without (gray shaded peak) 25 μM amuvatinib, and secondary only control (dashed line).

To determine whether death of CD138+ cells was associated with an effect on the target, total and phosphorylated-MET (p-MET) expression levels were determined by flow cytometry. There were sufficient numbers of cells from patients 6 and 8 to perform this assessment. In both samples, MET phosphorylation was reduced on Tyr 1234/1235 in the CD138+ cells by 40% and 50%, respectively, as compared to the time-matched controls (Figure 3A and B). In contrast, there was no detectable level of p-MET in CD138– cells from patient 8 as compared to isotype control (Figure 3C). The lack of p-MET in CD138– cells likely explains why this population of cells was not affected by amuvatinib treatment in any of the eight patient samples.

### Effect of Amuvatinib on Growth Inhibition

**Figure 4:**
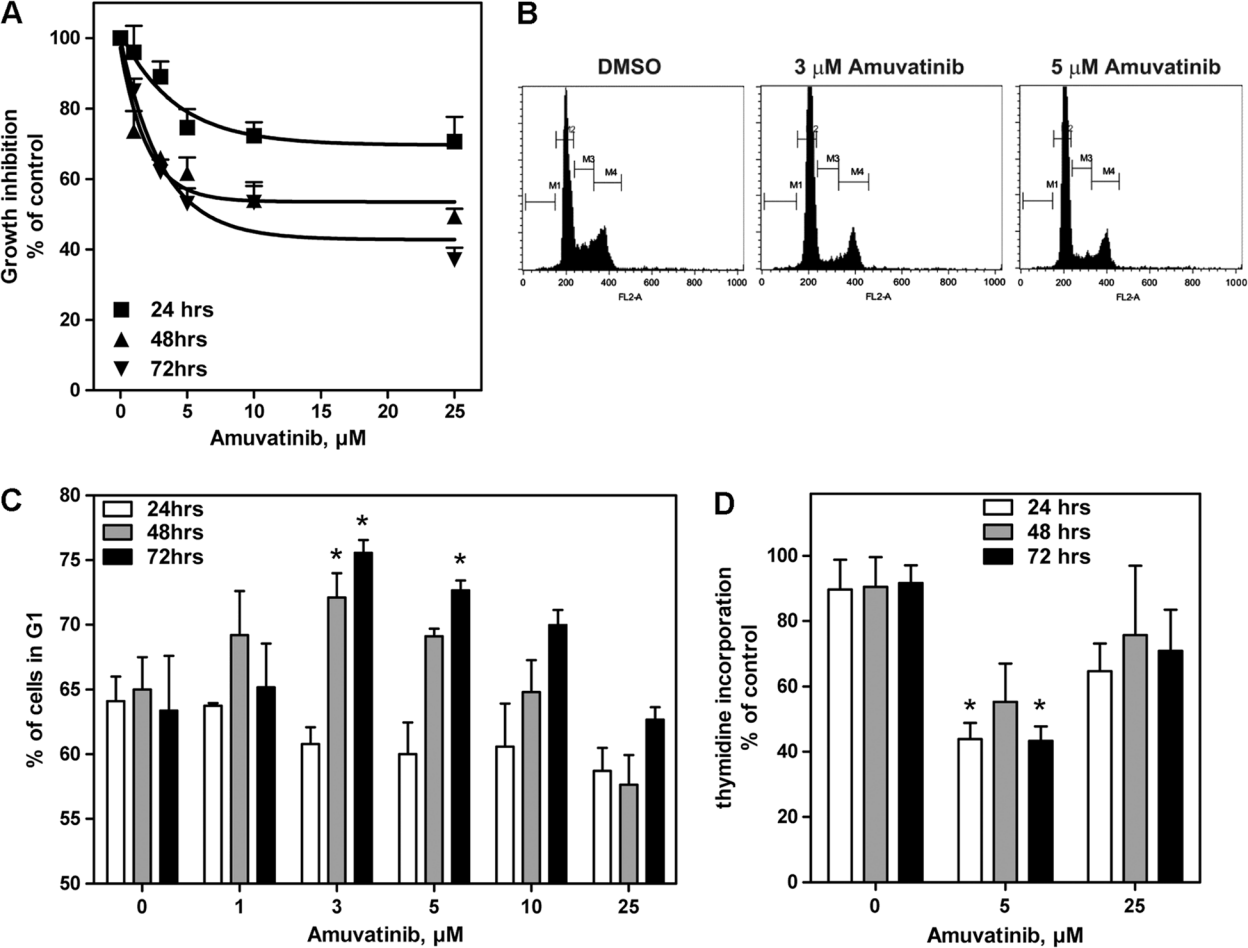
**Amuvatinib inhibits growth, induces cell cycle arrest, and inhibits DNA synthesis in myeloma cell line U266. (A)** U266 cells were treated with amuvatinib at various concentrations for 24 (■), 48 (▲), and 72 h (▼). Cells were counted using a Coulter counter, and the effect of the treatment on cell growth was determined. The data are presented in a time- and dose-dependent manner as percentages of time-matched controls. Data are representative of three independent experiments and presented as Mean ± SEM, n = 3, *P < 0.05. **(B)** To determine the effect of amuvatinib on the cell cycle, U266 cells were treated with 3 or 5 μM amuvatinib or DMSO for 72 h. Flow cytometry was used to assess the DNA content of cells stained with PI, regions labeled M1, M2, M3, and M4 represent cells in Sub-G_1_, G_1_, S, and G_2_/M phases of the cell cycle, respectively. **(C)** Cell cycle analyses were performed as in panel **(B)**, with treatments for 24 (white bars), 48 (gray bars), and 72 h (black bars) and the percentage of cells in the G_1_ phase of the cell cycle were plotted. Data are representative of three independent experiments and presented as Mean ± SEM, n = 3, *P < 0.05. **(D)** The effect of amuvatinib on DNA synthesis was determined by treating U266 cells with DMSO or 5 and 25 μM amuvatinib for 24 (white bars), 48 (gray bars), and 72 h (black bars) and quantifying thymidine incorporation. The data are presented as percentages of time-matched controls. Data are representative of three independent experiments and presented as Mean ± SEM, n = 3, *P < 0.05, **P < 0.01.

Since the number of the primary CD138+ cells in eight patient samples were insufficient for performing a detailed investigation of HGF/MET signaling, we decided to further investigate the effects of amuvatinib in a myeloma cell line. Because our results in the patient samples suggested that higher levels of HGF may be associated with an increased sensitivity to MET inhibition, we used the U266 cell line which expresses high levels of HGF [19]. Compared to DMSO-treated control cells, amuvatinib-treated U266 cells showed a dose- and time-dependent decrease in growth (Figure 4A). The growth inhibition was ~40% at a dose of 5 μM after 48 and 72 h of incubation and 50% at a dose of ~7 μM at 72 h.

### Effect of Amuvatinib on cell cycle arrest and DNA synthesis

We also tested whether the observed amuvatinib-induced growth inhibition was associated with an alteration in the cell cycle. At low micromolar doses (3 and 5 μM), U266 cells were arrested at G_1_ after 48 and 72 h (Figure 4B and C). In the DMSO-treated controls, approximately 65% of cells were in G_1_ phase at 72 h, while cells treated 3 μM amuvatinib significantly increased to 75% in G_1_ phase at the same time point (*p* < 0.05). Incubation with a higher level of amuvatinib (25 μM) resulted in a lower percentage of cells in G_1_ phase (Figure 4C), with a concomitant increase in the subG_1_ fraction (data not shown).

To determine whether the observed cell cycle changes were associated with an effect on DNA synthesis, we measured incorporation of thymidine in total DNA. Compared to DMSO-treated (control cells), amuvatinib-treated cells had decreased thymidine incorporation at doses of both 5 and 25 μM (Figure 4D), which was significantly higher for cells treated with 5 μM amuvatinib for 24 and 72 h, (*P* = 0.008 and *P* = 0.048, respectively). At 24 h, the inhibition of thymidine incorporation was greater than 50% with 5 μM amuvatinib. Additionally, as expected, the decrease in the cells’ S-phase DNA replicative capacity was discernible 24 h before there was a measurable change in the cell cycle profile as the doubling time for U266 cells is ~36 hours.

### Induction of Apoptosis by Amuvatinib

**Figure 5:**
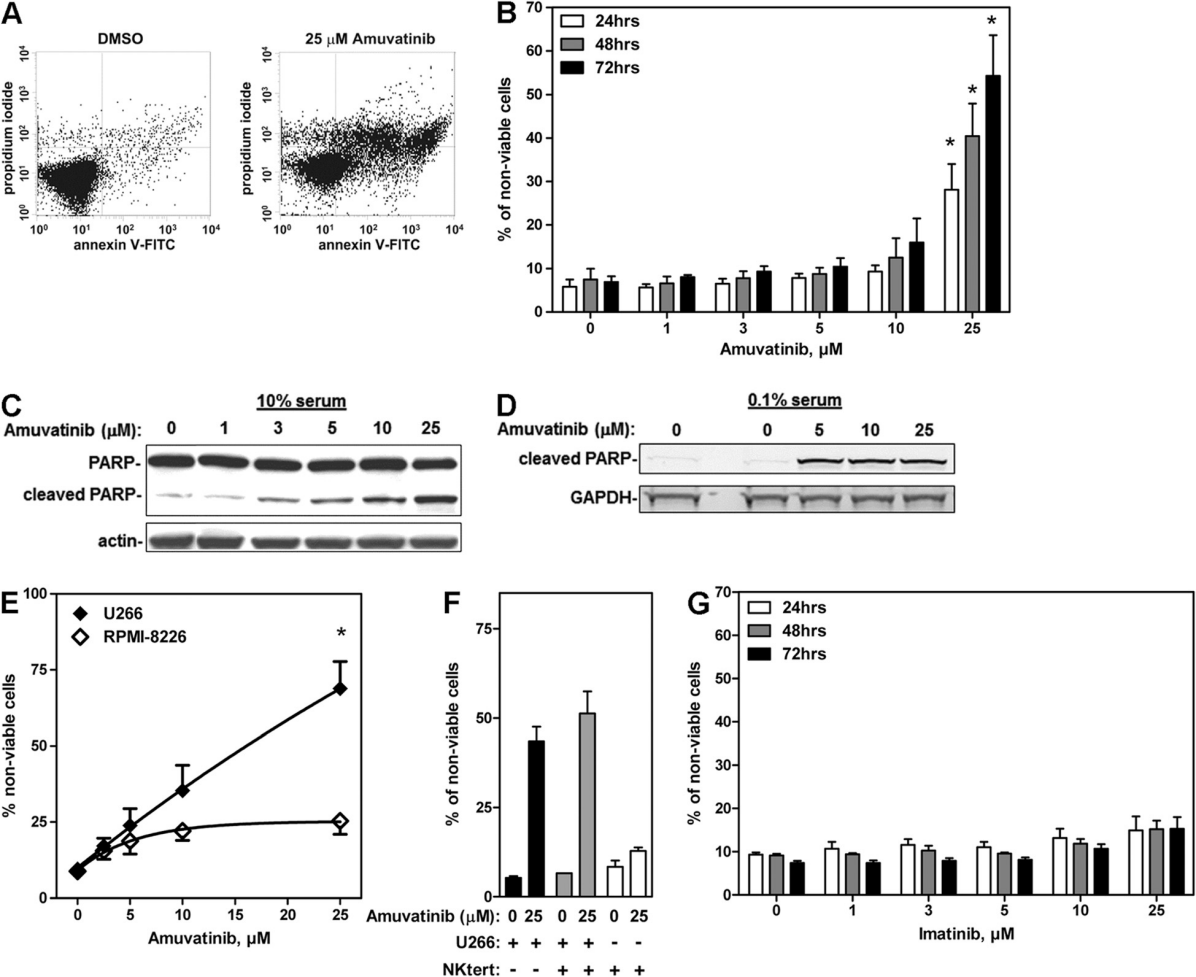
**Amuvatinib is tumoricidal to U266 myeloma cells.** Apoptosis was quantified by staining cells with annexin V/PI and measuring staining positivity using flow cytometry. **(A)** A representative annexin V/PI staining profile 72 h after treatment with 25 μM amuvatinib or DMSO is shown. **(B)** U266 cells were treated with DMSO or various concentrations of amuvatinib for 24 (white bars), 48 (gray bars), and 72 h (black bars) and percent of annexin V/PI positivity is presented. Data are representative of three independent experiments and presented as Mean ± SEM, n = 3, *P < 0.05. Immunoblot analysis demonstrating cleavage of PARP protein after treatment with amuvatinib at indicated concentrations under conditions of **(C)** full serum (10% FBS) for 24 h, or **(D)** serum starved conditions with endogenous HGF for 16 h, **(E)** U266 and RPMI-8226/S cells were treated with various concentrations of amuvatinib for 48 h and percent of annexin V/PI positivity is presented. Data are representative of three independent experiments and presented as Mean ± SEM, n = 3, *P < 0.05. **(F)** U266 cells cultured alone (black bars), or on NK-tert stromal cells (gray bars), or stromal cells alone (white bars), were treated with or without 25 μM amuvatinib for 48 h and assessed by flow cytometry for annexin V/PI staining. **(G)** Cells were treated with various concentrations of imatinib and annexin V/PI positivity is presented as percentage of time-matched controls. Data are representative of three independent experiments and presented as Mean ± SEM, n = 3, *P < 0.05.

Similar to what was seen in the primary CD138+ cells, U266 cells treated with 25 μM amuvatinib exhibited significantly greater cell death (*28*%*, 40*%*, and 54*%) than DMSO-treated controls (*6*%*, 7*%*, and 7*%) for 24, 48, and 72 h*,* respectively (Figure 5A and B) (*P* = *0.045*, *0.015*, and *0.018* respectively). This apoptotic induction was blocked by a pan-caspase inhibitor, ZVAD, suggesting a role for caspases in amuvatinib-mediated cell death (data not shown). Consistent with the annexin V/PI staining results was our finding that amuvatinib induced poly ADP ribose polymerase (PARP) cleavage in these cells in a dose-dependent manner. Under full serum conditions (10% fetal bovine serum (FBS)), an induction of PARP cleavage was seen after 24 h with doses as low as 3 μM amuvatinib with the highest level of cleaved PARP product was seen at 25 μM (Figure 5C).

Because 95% of amuvatinib binds serum proteins and is unavailable to the cells, we also examined the efficacy of amuvatinib under low serum (0.1% FBS) conditions. When cells were treated with 5 μM amuvatinib for 16 hours in the presence of low serum, but in the presence of endogenous autocrine HGF, we found maximum induction of PARP cleavage (Figure 5D). To further confirm the cytotoxic effects of amuvatinib in myeloma cells is associated with inhibiting the HGF/MET signaling axis, we compared the efficacy of amuvatinib to induce apoptosis in U266 cells versus RPMI-8226/S, a myeloma cell line which express 75% lower levels of HGF and 95% lower levels of MET (Additional file 1: Figure S1). As expected, there was a significant amuvatinib dose-dependent apoptosis-induction in the U266 cells after 48 h treatment (Figure 5E) (25 μM versus 0, 2.5, 5, and 10; *P* = *0.022, 0.018, 0.013, and 0.029,* respectively). In contrast there was only a minor apoptosis induction in the RPMI-8226/S cells which was not statistically significant (*P* = *0.053, 0.300, 0.427, and 0.503,* respectively). These results suggest that the apoptosis induction is due to targeting MET kinase which U266 cells are addicted to while RPMI-8226/S cells are not.

### Tumoricidal Effects of Amuvatinib in Myeloma Cells Grown in a Protective Stromal Environment

Bone marrow stroma provides a protective environment for MM cells [42]; thus, it is important to assess the efficacy of therapeutic agents in the context of a stromal environment. To assess this, we treated U266 cells co-cultured with and without stromal cells with amuvatinib for 48 h and measured viability by using flow cytometry analysis of annexin V/PI staining. Under these conditions, the U266 do not attach to the stromal cells, but are protected by them through both cell to cell contact and by various soluble factors produced by the stromal cells [43]. Amuvatinib induced ~50% cell killing during this time period and co-culture with the stromal cells provided no protection from this effect (Figure 5F). In contrast, these stromal cells were able to protect U266 cells from bortezomib treatment as they reduced the amount of bortezomib-induced apoptosis from ~75% to ~40% (Additional file 1: Figure S2A). To determine whether amuvatinib had an effect on the survival of stromal cells, stromal cells cultured alone were treated with amuvatinib, harvested by trypsinization, and similarly assessed for viability. Interestingly, amuvatinib had a very minimal effect on the survival of this population of cells (Figure 5F), though they express MET (Additional file 1: Figure S2B). These results indicate that the tumoricidal action of amuvatinib was largely restricted to the U266 myeloma cells, whereas the stromal cells, which are not addicted to MET, are not affected by this inhibitor. Furthermore, the stromal cells were not able to protect the U266 cells from amuvatinib’s tumoricidal activity.

Since amuvatinib also inhibits PDGFR and KIT, we validated MET kinase inhibition as the primary cause of cell death by using imatinib as a negative control. In addition to ABL, imatinib is known to also inhibit PDGFR and KIT but not MET [44]. In contrast to amuvatinib, 25 μM imatinib did not induce significant cell death (Figure 5G; *P* = 0.07) indicating that amuvatinib-mediated cell death is not due to its effects on PDGFR and KIT.

### Effect of Amuvatinib on MET Protein

**Figure 6:**
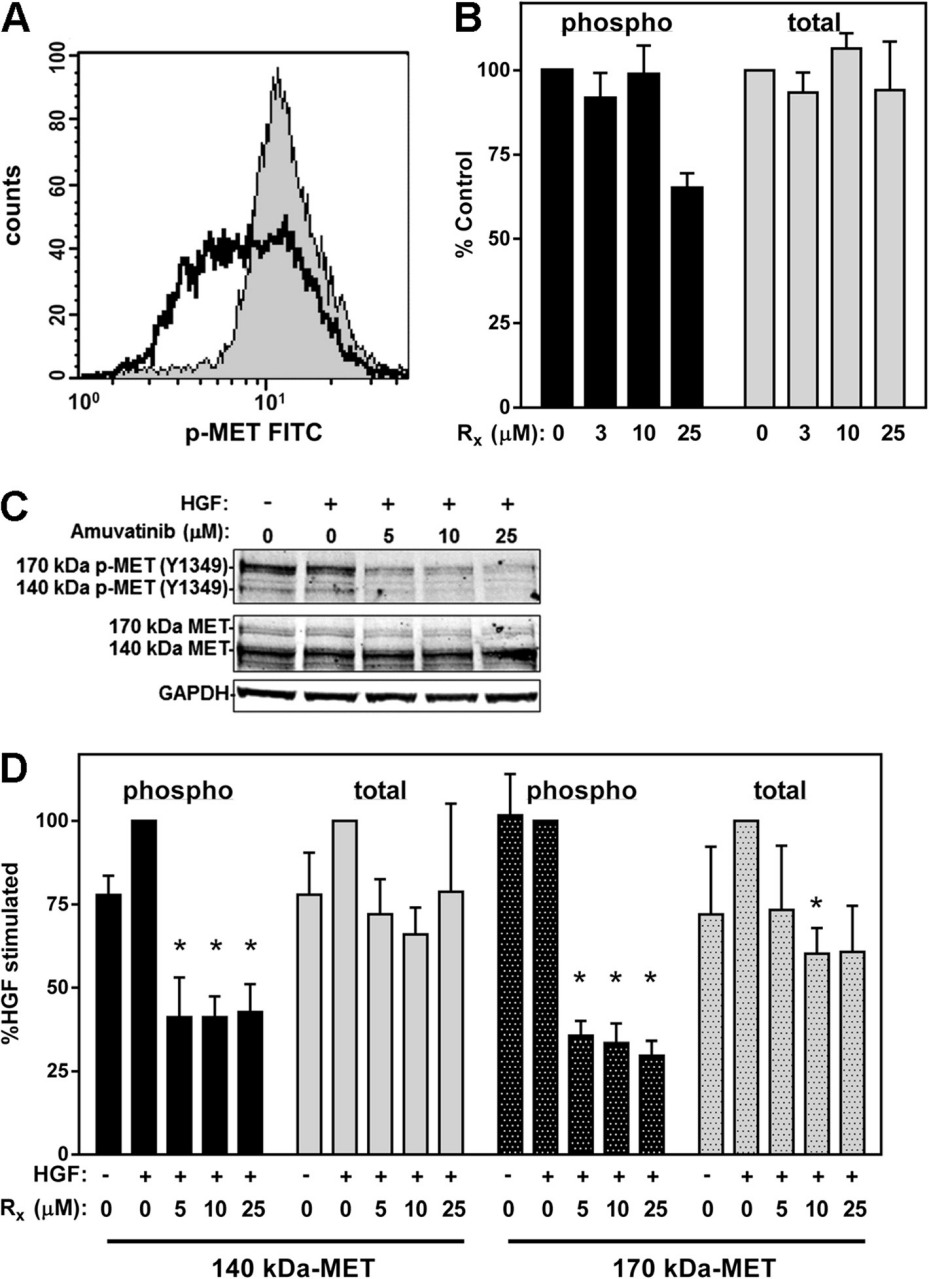
**Amuvatinib suppresses MET receptor tyrosine kinase activity. (A)** Flow cytometry analysis of p-MET (Tyr1234/1235) levels in U266 cells were treated for 24 hrs with DMSO (gray shaded) and 25 μM amuvatinib (dark black line). **(B)** Quantification of phospho (black bars) and total (gray bars) MET staining in U266 cells treated with the indicated concentrations of amuvatinib (Rx) from quadruplet experiments as in **(A)**. **(C)** U266 cells were serum starved and treated with the indicated concentrations of amuvatinib or DMSO and stimulated with 50 ng/ml HGF for 15 min. Cell lysates were subjected to immunoblot analysis to assess MET (Y1349) phosphorylation. **(D)** The 140 kDa (solid bars) and the 170 kDa (speckled bars) phospho (black bars) and total (gray bars) MET bands from triplicate experiment as in **(C)** were quantitated and normalized to GAPDH levels. The results are presented as percentages of the HGF-stimulated DMSO controls. Data are representative of three independent experiments and presented as Mean ± SEM, n = 3, *P < 0.05, **P < 0.01.

To further investigate the effect of amuvatinib on MET signaling, we first measured MET receptor tyrosine kinase activity in U266 cells by flow cytometry. Similar to the results seen with the CD138+ cells from the patient samples, a reduction in MET phosphorylation was detected in cells treated for 24 hr with 25 μM amuvatinib when cells are grown in normal growth conditions (10% FBS) (Figure 6A and B). This decrease of p-MET was associated with cell death as cell death was induced with 25 μM amuvatinib when cells are grown in normal growth conditions (10% FBS) (Figure 5B).

To assess the effects of amuvatinib on HGF-specific signaling, protein lysates from U266 cells serum starved in 0.1% FBS for 16 h with and without various concentrations of amuvatinib followed with 15 min HGF stimulation were examined by immunoblot analysis. The results showed that under serum starved conditions, treatment with 5 μM amuvatinib, decreased phosphorylation of the processed ~140 kDa MET β-chain at Tyr1349 by ~60% (Figure 6C and D). Because of the autocrine stimulation of MET by the endogenous HGF produced in these cells, MET was phosphorylated under serum-starved conditions even without the addition of exogenous HGF. Furthermore, an amuvatinib-dependent decrease of total MET levels of ~30% was also observed. A ~170 kDa phosphorylated MET band was detected at ~2 fold higher levels than the 140 kDa band in untreated U266 cells. A comparison with total MET shows both bands were present but the levels of the total 140 kDa band was ~4 times greater than the levels of the 170 kDa band. Although unprocessed pro-MET, containing both the α and β subunits, has been detected by SDS PAGE as a 170 kDa band, it has not been associated with kinase activity. Conversely, a splice variant of *MET* containing an additional 54 nt of exon 10 has been reported to be expressed at low levels [45]. This splice form produces a MET isoform that has kinase activity, though it cannot be processed into α and β subunits. In U266 cells, amuvatinib inhibited phosphorylation of a 170 kDa MET by ~70% (Figure 6C and D, B). Again, the decrease of HGF-specific phosphorylation of both isoforms of MET under low serum conditions is associated with cell death under low serum conditions (Figure 5D). The lower concentration of amuvatinib needed to decrease MET phosphorylation under 0.1% serum versus 10% serum conditions is in agreement with binding of the drug by serum proteins. Additionally, the concentration of amuvatinib required to decrease MET phosphorylation correlates with the concentration required to induce cell killing under either growth conditions. These results suggest the amuvatinib-induced cell death was associated with reduced MET activity.

### Effect of Amuvatinib on downstream targets of MET

**Figure 7:**
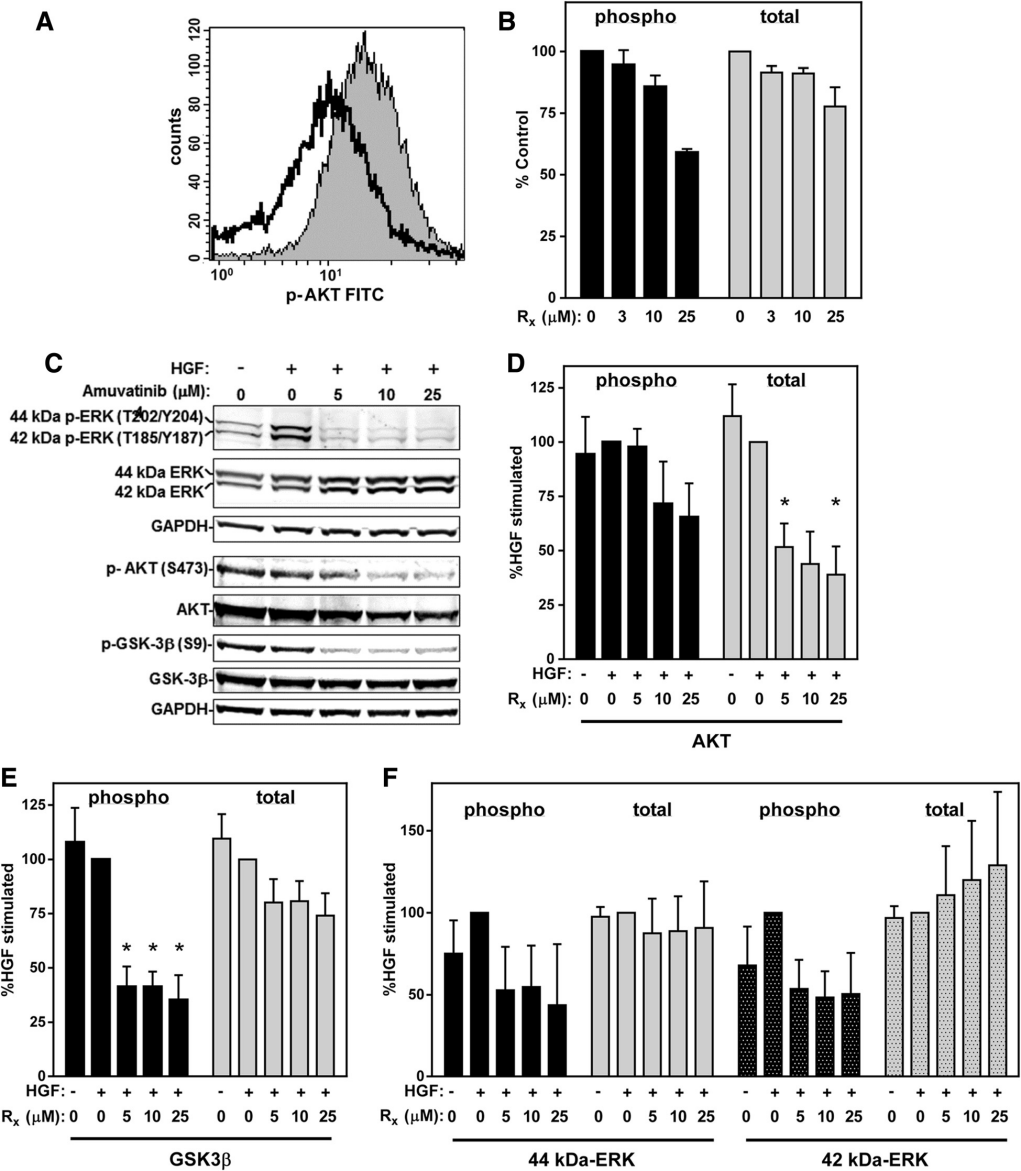
**Amuvatinib inhibits MET signaling. (A)** Flow cytometry analysis of p-AKT (Ser473) levels in U266 cells were treated for 24 hrs with DMSO (gray shaded) and 25 μM amuvatinib (dark black line). **(B)** Quantification of phospho (black bars) and total (gray bars) AKT staining in U266 cells treated with the indicated concentrations of amuvatinib (Rx) from five experiments as in **(A)**. **(C)** U266 cells were serum starved, treated with the indicated concentrations of amuvatinib or DMSO, and stimulated with 50 ng/ml HGF for 15 min. Cell lysates were subjected to immunoblot analysis to assess phosphorylation of ERK p44/42, AKT, and GSK-3β. AKT **(D)**, GSK-3β **(E)**, and ERK p44 (solid bars) and p42 (speckled bars) **(F)**, phospho (black bars) and total (gray bars) bands were quantitated and normalized to GAPDH levels. The results are presented as a percentage of the HGF stimulated DMSO control. Data are representative of three independent experiments and presented as Mean ± SEM, n = 3, *P < 0.05.

Previous studies have shown that inhibition of MET causes a reduction in the phosphorylation of both AKT and extracellular signal-regulated kinases (ERK)1/2 in the MAPK signaling pathway [11]. The regulation of AKT activity by MET plays a prominent role in promoting cell survival. Moreover, MET regulation of the ERK pathway is important for proliferation and both the ERK1/2 and AKT pathways are involved in MET-induced cell spreading and motility. To examine AKT activity, p-AKT (S473) levels were measured in U266 cells by flow cytometry. Similar to the results seen with p-MET, a reduction in AKT phosphorylation was detected in cells treated for 24 hr with 25 μM amuvatinib when cells were grown in normal growth conditions (10% FBS) (Figure 7A and B). An assessment of amuvatinib’s effects on HGF-specific signaling was also performed in the U266 cells cultured in 0.1% FBS for 16 h with and without various concentrations of amuvatinib followed with 15 min HGF. Immunoblot analysis again showed that lower concentrations amuvatinib is needed to decreased AKT phosphorylation at Ser473 (Figure 7C and D), even though in these cells the levels were low and difficult to detect. Interestingly, total AKT decreased by 60% with amuvatinib treatment. To better assess the effect of amuvatinib on the AKT pathway, we examined the phosphorylation of an AKT target, glycogen synthase kinase 3 β (GSK3β) on Ser9. Amuvatinib-treated cells showed, in addition to reduction of AKT, a 65% decrease in phosphorylation of GSK3β, with a 24% decrease in total GSK3β (Figure 7C and E).

A similar assessment of phospho-ERK1/2 levels under HGF specific signaling demonstrated that amuvatinib inhibited phosphorylation of both the 44-kDa and the 42-kDa ERK isoforms by 55% and 50%, respectively, while total ERK1/2 levels did not significantly change (Figure 7C and F). These results demonstrate that amuvatinib treatment inhibits both ERK1/2 and AKT signaling through the MET pathway.

## Discussion

MET is a receptor tyrosine kinase that is activated by the ligand HGF and has been shown to be constitutively expressed, mutated, or over-expressed in many different cancer cell types. It serves as an important factor for cell survival, migration, and motility [7,11,15]. Corollary to that, inhibition of MET kinase activity causes reduction of the downstream signaling that is necessary for these cells to maintain their oncogenic properties [46]. Previous studies in our laboratory showed that while MET receptor tyrosine kinase acts as a survival factor for myeloma cells [32,33], it is neither mutated nor, for the most part, over-expressed in MM. However, its ligand HGF is increased in plasma or serum obtained from myeloma patients and higher HGF level has been associated with poor prognosis [18,20,22,26]. Furthermore, HGF not only promotes growth, migration, and survival of myeloma cells, it also potentiates IL-6 effects [46].

While levels of plasma HGF have been associated with myeloma, levels of *HGF* and *MET mRNA* in patient plasma cells have not been well evaluated nor correlated with disease status. Our analyses of mRNA array data [40,41] demonstrated autocrine expression of HGF in CD138+ plasma cells from MM patients. This was consistent with previous report in 7 myeloma patient samples [18]. Our results further elucidated that the level of the *HGF* expression was directly associated with disease progression.

Together, these findings provide a rationale for targeting the HGF/MET signaling axis in myeloma. Targeting HGF directly may prove difficult, since therapeutic targeting of HGF would need to be effective at elevated levels to successfully compete and inhibit the high serum HGF concentrations in myeloma patients. Therefore, using a small-molecule MET suppressor such as amuvatinib may be a viable option to target the HGF/MET pathway. Additionally, several MET inhibitors are available for clinical testing [11].

Amuvatinib is an orally available drug that is currently in clinical trials for the treatment of solid tumors [37,38,47]. This compound was designed, developed, and selected via a computation-driven *in silico* process whereby drug scaffolds were screened, docked, and fitted against a homologous model of KIT. After additional screening in biochemical and cell-based assays, amuvatinib was selected as a tyrosine kinase inhibitor with activity against wild-type and mutant KIT, MET, RET, FLT3 and PDGFRα [48,49]. Later, amuvatinib inhibition of MET activity was found to lead to reduction of RAD51 expression and to radiosensitization of tumor cells [50].

Since amuvatinib is a small-molecule inhibitor that suppresses MET activity, we tested this agent as a proof-of-concept to therapeutically target MET in myeloma. Our study demonstrated that amuvatinib was effective in inhibiting growth and DNA synthesis at low micromolar concentrations in cell lines grown under normal conditions (10% FBS). Moreover, amuvatinib treatment resulted in cell death in U266 myeloma cell line dependent on MET/HGF signaling, as measured by annexin V/PI staining and PARP cleavage. This cytotoxic effect remained even when these MET-addicted cells were grown on bone marrow stromal cells. In contrast, the drug did not induce apoptosis in another myeloma cell line (RPMI-8226/S) that is not dependent on the MET/HGF signaling axis due to lower levels of *HGF* (75% less) and *MET* (95% less).

Because amuvatinib also impairs KIT and PDGFR signaling, we tested impact of imatinib (an established KIT and PDGFR inhibitor) in myeloma cells. Imatinib induced no significant amount of cell death in U266 cells demonstrating that amuvatinib’s effect was due to MET inhibition. This statement was in line with the data regarding decreased phosphorylation of MET after amuvatinib treatment. Because >95% of the compound is bound and sequestered by serum proteins (Unpublished data), the dose required to achieve maximum inhibition of MET phosphorylation in serum starved conditions was lower than the dose to induce apoptosis in full serum conditions. Likewise, under serum starved conditions, the maximum induction of apoptosis was seen at the same dose which achieved maximum inhibition of MET phosphorylation. As expected, in imatinib treated cells, there was no reduction of p-MET (data not shown) as well as no significant reduction in survival. These correlation data suggest that amuvatinib mediated growth inhibition and cell death is due to its action on MET and not its action on KIT or PDGFR.

In conjunction with a decrease in MET phosphorylation, there was a decline in HGF-dependent ERK1/2 and AKT phosphorylation as well as the phosphorylation of the AKT targets GSK3β and caspase-9 (data not shown). Diminution of phosphorylated MET and associated decreases in ERK1/2 and AKT phosphorylation has been shown to be important in growth, migration and cell survival pathways for other cancer cell types [11].

Amuvatinib proved to be effective in inducing cell death not only in a MET dependent myeloma cell line but also in primary CD138+ malignant plasma cells obtained from patients with myeloma. In contrast, amuvatinib did not cause cell death in normal CD138– cells obtained from the same individuals (Figure 2). These data provide evidence of the selectivity of amuvatinib, suggesting that it may be used specifically for myeloma treatment without impairing other normal hematological cells in the bone marrow. In line with this selective cytotoxic effect on CD138 plasma cells, MET phosphorylation was reduced by amuvatinib treatment in primary plasma cells but not CD138– cells.

The effects of amuvatinib described here provide proof-of-concept that MET is important for the survival of myeloma cells and that reduction of its kinase activity may prove to be an effective targeted therapy. The 25 μM dose of amuvatinib needed to robustly induce apoptosis in cell lines and plasma cells under full serum conditions may not be achievable *in vivo*. Pharmacokinetic studies of amuvatinib during a phase I trial indicated that plasma levels reached between 1 and 2 μM [51]. Hence, newer generation and more potent MET tyrosine kinase inhibitors are needed [11]. ARQ 197 (tivantinib) is a small-molecule, non-ATP-competitive inhibitor which is highly specific for MET [52,53]. This drug is well tolerated in clinical trials and has shown efficacy in solid tumors [54-58]. Pharmacodynamic studies from a phase I trial indicated that at an oral dosing of 360 mg, twice daily, ARQ 197 reached steady-state plasma concentrations of 6–7 μM [55]. This correlated with decreases in total MET and phospho-FAK (Tyr861) and increases in TUNEL-positive cells in patients’ tumors.

Our results with amuvatinib provided the impetus to pursue testing of ARQ 197 in myeloma cells. Our preclinical studies indicated that treatment with ARQ 197 for 48 hours was cytotoxic to myeloma cell lines (≥ 60% increase in annexin V/PI-positive cells) at clinically achievable doses [59]. Moreover, these studies provided the foundation for a Cancer Therapy Evaluation Program, National Cancer Institute sponsored phase 2 clinical trial of ARQ 197 in myeloma patients, which is currently underway at MD Anderson Cancer Center [60].

## Conclusions

Our finding provides proof-of-principle that MET is important for the survival of myeloma cells and using a MET inhibitor such as amuvatinib may prove to be an effective strategy for treatment of MM. Amuvatinib exhibited tumoricidal activity in myeloma cells which was associated with inhibition of MET signaling. Amuvatinib’s lack of effect on CD138– cells from the same patients further establishes the selectivity of this agent. The clinical success of other targeted therapeutics for cytoplasmic and receptor tyrosine kinases, further underscores a need for testing a small-molecule inhibitor that targets MET kinase activity for patients with myeloma.

## Methods

### Materials

Amuvatinib (MP470) was obtained from Astex Pharmaceuticals, Inc. (Dublin, CA) and was dissolved in DMSO (Sigma Aldrich, St. Louis, MO). Because of stability constraints, amuvatinib solution was prepared fresh for each experiment. Imatinib was purchased from Novartis (St. Louis, MO) and was dissolved in DMSO and stored in aliquots at -20°C. [^3^H] thymidine (60 Ci/mmol) was obtained from Moravek Biochemical Inc. (Brea, CA).

### Cell culture and growth analysis

The myeloma cell lines U266 [61] and RPMI-8226/S [62] were obtained from Dr. William Dalton at H. Lee Moffitt Cancer Center (Tampa, FL). NK-tert human bone marrow stromal cells were obtained from Dr. Jan Burger at UT MD Anderson Cancer Center [63]. The cell lines were maintained as described [32,63] and routinely tested for *Mycoplasma* infection and authenticated by short tandem repeat analysis by UT MD Anderson Cancer Center’s Characterized Cell Line Core facility.

Myeloma cell-stromal co-cultures were performed using U266 cells and NK-tert cells at a ratio of 20 to 1. Stromal cells were plated at a concentration of ~2 × 10^2^ cells/mm^2^ surface area 5 hours before adding U266 cells at a 20 fold higher concentration. The cells were co-cultured for 2 h prior to treatment with or without amuvatinib or bortezomib for 48 h. At the end of incubation, the U266 cells, which are free floating in these cultures, were carefully removed for analysis, leaving the adherent stromal layer undisturbed. Additionally, the stromal cells were also harvested by trypsinization and similarly assessed.

The effect of amuvatinib treatment on cell growth inhibition was measured in exponentially growing U266 cells. Cells were counted using a Coulter counter (Beckman Coulter, Fullerton, CA). DNA synthesis was measured using [^3^H]thymidine incorporation as described [64].

### Gene expression array analyses

Expression data from 162 CD138+ bone marrow plasma cell samples from healthy individuals as well as patients with MGUS, SMM, MM-N, and MM-R, which were measured by using Affymetrix U133A microarrays, were downloaded from GEO (GSE6477) [40,41]. Robust Multichip Average (RMA) algorithm was used for normalization/quantification of the data. The maximal values for the respective probe-sets of *MET* and *HGF* were used for gene expression profiling. The Kruskal-Wallis test was applied to assess whether expression of *MET* and *HGF* was associated with defined clinical groups, and results are presented as box-plots.

### Isolation of CD138+ AND CD138– cells from primary bone marrow aspirates from MM patients

Primary samples were obtained from both male and female myeloma patients being treated at MD Anderson Cancer Center (Table 1). Patient samples were obtained using an MD Anderson Cancer Center Institutional Review Board approved protocol. All patients signed an informed consent form to provide peripheral blood and bone marrow samples. After collection of bone marrow samples, CD138+ cells were isolated as described [65], suspended in RPMI 1640 with 10% human AB serum (Cambrex Biosciences, East Rutherford, NJ) and used immediately for experiments. Peripheral blood samples were collected from patients 2, 4, and 5 for assessment of plasma HGF levels.

### Immunoblot analysis

The effects of amuvatinib on HGF dependent signaling were assessed in U266 cells that had been serum starved for 24 h in RPMI 1640 containing 0.1% FBS; for the last 16 h of starvation the cells were treated with various concentrations of amuvatinib or DMSO. They were then treated with 50 ng/ml HGF for 15 min to stimulate MET. Amuvatinib-mediated induction of PARP cleavage was performed on U266 cells cultured in full serum (10% FBS) as well as under low serum conditions (0.1% FBS). Protein lysates and immunoblots were prepared as previously described [66]. Experiments were performed in triplicates, and bands were quantified by using an Odyssey Infrared Imaging System (LI-COR Biosciences, Lincoln, NE). Primary antibodies were: mouse monoclonal antibodies to MET clone 3D4 (Invitrogen, Carlsbad, CA); GSK-3β clone 7/GSK-3b, PARP clone C2-10, cleaved PARP Asp 214 clone F21-852, AKT clone 9Q7 (BD Biosciences Pharmingen, San Diego, CA); GAPDH clone 6C6 (Abcam, Inc, Cambridge, MA); phospho-ERK1/2 (Thr202/Tyr204) clone E10 (Cell Signaling Technology, Danvers, MA); β-actin clone AC-15 (Sigma Aldrich); rabbit monoclonal antibodies to phospho-GSK-3β (Ser9) clone 5B3 (Cell Signaling Technology); rabbit polyclonal antibodies to phospho-MET (Tyr1349) (Invitrogen); ERK1/2, and phospho-AKT (Ser473) (Cell Signaling Technology).

### Flow cytometry

Intracellular protein expression in U266 cells was measured using BD Cytofix/Cytoperm Fixation/Permeabilization Kit (BD Biosciences). Primary antibodies used were anti-phospho-HGF R/c-MET (Tyr1234/1235) (R&D Systems, Minneapolis, MN), MET (C-12): sc-10 (Santa Cruz Biotechnology, Santa Cruz, CA), phospho-AKT (Ser473), AKT antibody (Cell Signaling Technology); and caspase-9 (Ser196) (Santa Cruz Biotechnology). Secondary antibody was a fluorescein isothiocyanate–conjugated (FITC) Affinipure goat anti-rabbit (Jackson ImmunoResearch, West Grove, PA). Cell cycle analysis and annexin V/propidium iodide (PI) staining were performed, respectively, as described [32,33]. All flow cytometry analysis was performed using a Becton Dickinson FACSCalibur flow cytometer (San Jose, CA, USA). Statistical significance of changes was assessed by paired *t*-test analysis using Prism software (Graphpad, San Diego, CA).

### Enzyme linked-immuno-sorbent assay for HGF levels

HGF levels in primary patient plasma were determined using the Human HGF Immunoassay Kit as per the manufacturer’s protocol (Invitrogen). The absorbance of this horseradish-peroxidase based assay was measured at 450 nm. Each sample was assayed in triplicate.

## Abbreviations

DMSO: Dimethyl sulfoxide; ERK: Extracellular signal-regulated kinases; FBS: Fetal bovine serum; GSK3β: Glycogen synthase kinase 3 β; HGF: Hepatocyte growth factor; IL-6: Interleukin-6; MAPK: Mitogen-activated protein kinase; MGUS: Monoclonal gammopathy of undetermined significance; MM: Multiple myeloma; MM-N: Newly-diagnosed multiple myeloma; MM-R: Relapsed/refractory disease; PARP: Poly ADP ribose polymerase; PDGFR: Platelet derived growth factor receptor; PI: Propidium iodide; SMM: Smoldering multiple myeloma.

## Competing interest

SR and PT are employed by Astex Pharmaceuticals, Inc., Dublin, CA. For the remaining authors, none was declared.

## Authors’ contributions

CJP, SZ, KB, and CMS designed and performed experiments; CJP, SZ, JZ, VB, and CMS analyzed data; SS performed experiments; PT and SR provided amuvatinib; MW provide sorted bone marrow samples; CJP and CMS wrote the manuscript; CMS and VG directed study; VG provided laboratory resources; All authors critically read and approved manuscript.

## Supplementary Material

Additional file 1 Figure S1 MET and HGF expression. Real-time RT-PCR analysis of HGF and MET transcript levels in U266 and RPMI-8226/S cells. **Figure S2.** (A) NK-tert cells protect U266 cells from bortezomib-induced cytotoxicity. (B) MET expression and HGF-dependent activity in serum starved NK-tert cells. Click here for file
