# Erratum to: Targeting MET kinase with the small-molecule inhibitor amuvatinib induces cytotoxicity in primary myeloma cells and cell lines

**DOI:** 10.1186/s13045-016-0335-5

**Published:** 2016-10-13

**Authors:** Cornel Joseph Phillip, Shadia Zaman, Shujun Shentu, Kumudha Balakrishnan, Jiexin Zhang, Veera Baladandayuthapani, Pietro Taverna, Sanjeev Redkar, Michael Wang, Christine Marie Stellrecht, Varsha Gandhi

**Affiliations:** 1 Departments of Experimental Therapeutics, The University of Texas MD Anderson Cancer Center, Houston, Texas USA; 2 Bioinformatics and Computational Biology, The University of Texas MD Anderson Cancer Center, Houston, Texas USA; 3 Biostatistics, The University of Texas MD Anderson Cancer Center, Houston, Texas USA; 4 Leukemia, The University of Texas MD Anderson Cancer Center, Houston, Texas USA; 5 Lymphoma/Myeloma, The University of Texas MD Anderson Cancer Center, Houston, Texas USA; 6 Graduate School of Biomedical Sciences, The University of Texas Health Science Center, Houston, Texas USA; 7 Astex Pharmaceuticals, Inc., Dublin, California USA

## Erratum

After the publication of this work [1], it was brought to our attention that two points needed to be explained further.

The first point was regarding the GAPDH immunoblot in Fig. 6c. We had performed this experiment in triplicate. The images used for phospho- and total MET in Fig. 6c were derived from the third replicate of the experiment, but accidentally we used the GAPDH from the second replicate of the experiment.

**Fig. 6:**
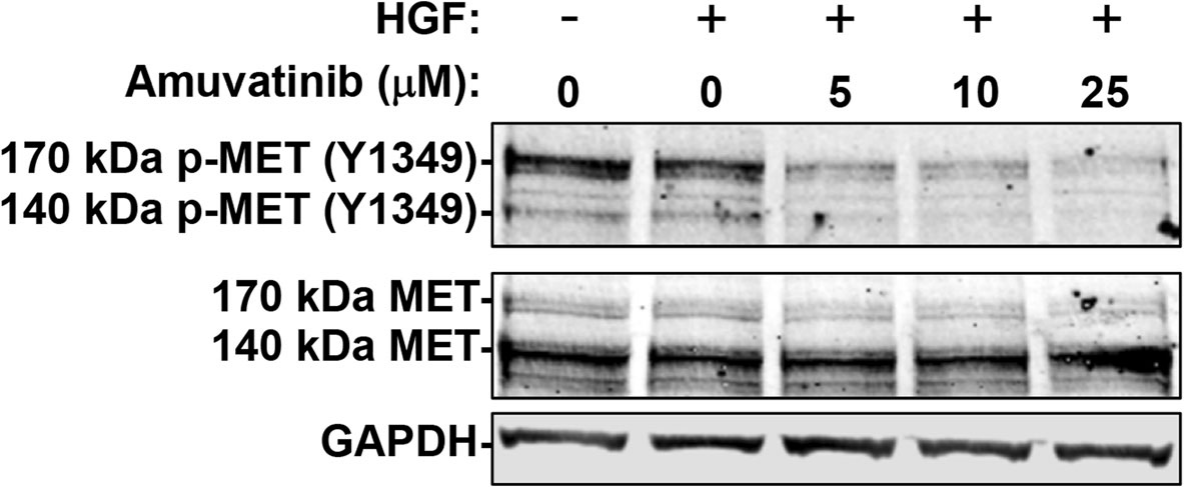
U266 cells were serum starved and treated with the indicated concentrations of amuvatinib or DMSO and stimulated with 50 ng/ml HGF for 15 min. Cell lysates were subjected to immunoblot analysis. After transfer, the membrane was cut around 80 kDa and top portion was used for this figure while lower (<80 kDa was used for GAPDH as well as quantitation of proteins in Fig. 7d and e). To assess MET (Y1349) phosphorylation and total MET, the upper portion of the membrane was first probed with a rabbit anti-phospho-MET (Y1349) antibody followed by the secondary, which was a green fluorescent anti-rabbit antibody. Next, we probed with a mouse anti-total-MET antibody followed by the secondary, which was a red fluorescent anti-mouse antibody. The last antibody, the bottom portion of the membrane was probed with, was a mouse GAPDH antibody followed by the secondary red fluorescent anti-mouse antibody. We used a fluorescence based imaging system, LiCor Odyssey. The Odyssey software was used to convert color image to grey scale image for publication

The revised Fig. 6c can be seen at the end of this Erratum with the corresponding and correct GAPDH image.

The interpretation of the results and findings of our investigations remain unchanged. As such, the results section that describes Fig. 6c remains the same. Also, correct images were used for quantification of the bands, and hence the quantitation figure (Fig. 6d) and associated text also do not change. Our findings remain valid and our inadvertent error did not affect our results, discussion, or the conclusions from this work, as the text refers to the correct values. We sincerely regret our error and apologize for any confusion this may have caused.

The second point was regarding similarity in the GAPDH and GSK-3β immunoblot (last 2 rows) in Fig. 7c. To generate this figure, protein lysates from each experiment were run on two different gels and transferred. The membranes from both gels were cut based on molecular marker, so that we would have a piece of the immunoblot containing proteins above ~80 kDa and a second piece containing proteins below ~80 kDa. The pieces of membranes above ~80 kDa were probed for Total and phospho-Met (quantitation of these blots were used in Fig. 6). Lower portions were used for Fig. 7c. One was used for phospho- and total-ERK and corresponding GAPDH. The other was used for phospho and total AKT (around 60 kDa) and phospho and total GSK-3β (around 40 kDa), along with GAPDH (around 37 kDa).

The lower 5 rows of the immunoblot were all from the same membrane and were probed as below. The membrane was first probed with a rabbit anti-phospho-AKT (S473) antibody followed by the secondary, which was a green fluorescent anti-rabbit antibody. Next, we probed with a mouse anti-total-AKT antibody followed by the secondary, which was a red fluorescent anti-mouse antibody. Next, we probed the blot with a rabbit anti-phospho-GSK-3β (S9) antibody followed by the secondary, which was a green fluorescent anti-rabbit antibody. Next, we probed with a mouse anti-total-GSK-3β antibody followed by the secondary, which was a red fluorescent anti-mouse antibody. Lastly, we probed with a mouse GAPDH antibody followed by the secondary red fluorescent anti-mouse antibody. We use a fluorescence based imaging system, LiCor Odyssey, to analyze immunoblots. With this system, our secondary antibodies against primary antibodies derived from different species are tagged with different fluorescence colors. The Odyssey software allows us to image each fluorescent channel separately as well and convert color image to grey scale image for publication. Because all 5 rows were from the same gel, and GSK3beta is fairly close to GAPDH, the shapes of each band in these two proteins appear similar. However, they are separate immunogenic reacted proteins as described above. This experiment was done in biological triplicate and quantitated using Odyssey Imaging software. The quantitated values were graphed in Fig. 7d, 7e, and 7f of the same published paper.

Because there were no errors in this figure, the published Fig. 7c is correct.
