# Supplementary figures and images for: Targeting MET kinase with the small-molecule inhibitor amuvatinib induces cytotoxicity in primary myeloma cells and cell lines
Source: J Hematol Oncol. 2013 Dec 10;6:92. doi: 10.1186/1756-8722-6-92 (PMC3878866; doi:10.1186/1756-8722-6-92)

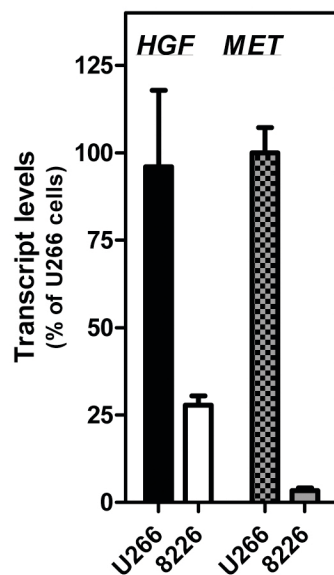

**Supplemental Figure 1**

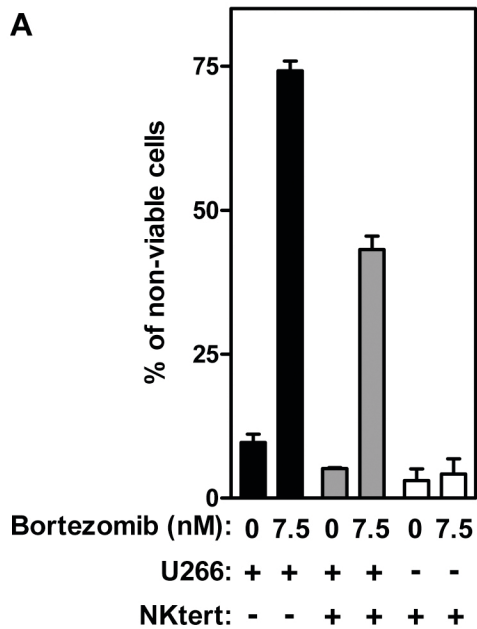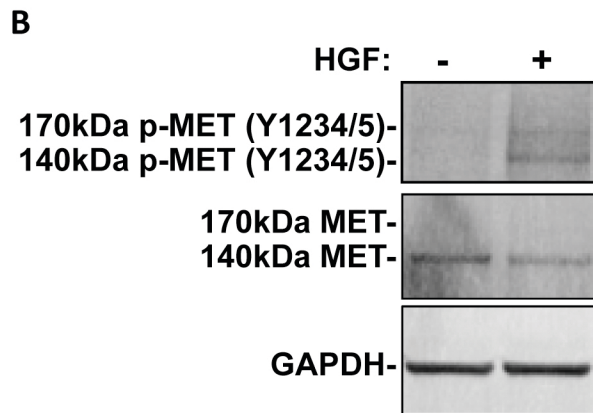

**Supplemental Figure 2**

Supplement: Additional file 1 Figure S1 — MET and HGF expression. Real-time RT-PCR analysis of HGF and MET transcript levels in U266 and RPMI-8226/S cells. Figure S2. (A) NK-tert cells protect U266 cells from bortezomib-induced cytotoxicity. (B) MET expression and HGF-dependent activity in serum starved NK-tert cells. [file 1756-8722-6-92-S1.pdf]
